# *In Vivo* Virulence Characterization of Pregnancy-Associated Listeria monocytogenes Infections

**DOI:** 10.1128/IAI.00397-18

**Published:** 2018-10-25

**Authors:** Holly A. Morrison, David Lowe, Jennifer R. Robbins, Anna I. Bakardjiev

**Affiliations:** a Benioff Children's Hospital, Microbial Pathogenesis and Host Defense Program, University of California, San Francisco, San Francisco, California, USA; b Department of Biology, Xavier University, Cincinnati, Ohio, USA; University of Illinois at Chicago

**Keywords:** DNA barcode, *Listeria*, clinical isolate, epidemiology, placental infection, placental pathogen, signature tag, virulence

## Abstract

Listeria monocytogenes is a foodborne pathogen that infects the placenta and can cause pregnancy complications. Listeriosis usually occurs as a sporadic infection, but large outbreaks are also reported. Virulence from clinical isolates is rarely analyzed due to the large number of animals required, but this knowledge could help guide the response to an outbreak. We implemented a DNA barcode system using signature tags that allowed us to efficiently assay variations in virulence across a large number of isolates. We tested 77 signature-tagged clones of clinical L. monocytogenes strains from 72 infected human placentas and 5 immunocompromised patients, all of which were isolated since 2000. These strains were tested for virulence in a modified competition assay in comparison to that of the laboratory strain 10403S. We used two *in vivo* models of listeriosis: the nonpregnant mouse and the pregnant guinea pig. Strains that were frequently found at a high abundance within infected organs were considered hypervirulent, while strains frequently found at a low abundance were considered hypovirulent. Virulence split relatively evenly among hypovirulent strains, hypervirulent strains, and strains as virulent as 10403S. The laboratory strain was found to have an intermediate virulence phenotype, supporting its suitability for use in pathogenesis studies. Further, we found that splenic virulence and placental virulence are closely linked in both the guinea pig and mouse models. This suggests that outbreak and sporadic pregnancy-associated L. monocytogenes strains are not generally more virulent than lab reference strains. However, some strains did show consistent and reproducible virulence differences, suggesting that their further study may reveal deeper insights into the biological underpinnings of listeriosis.

## Abstract (precis)

Listeria monocytogenes is a foodborne pathogen that infects the placenta and can cause pregnancy complications. Listeriosis usually occurs as a sporadic infection, but large outbreaks are also reported.

## INTRODUCTION

Listeriosis is a foodborne disease that afflicts humans worldwide (1, 2). In the United States, the Centers for Disease Control and Prevention (CDC) estimates that it is responsible for approximately 1,600 cases and 260 deaths per year (3). Most cases occur in predisposed individuals, such as immunocompromised patients, neonates, and elderly adults. In those cases, the main clinical manifestations are sepsis, meningoencephalitis, and death (4). With a mortality rate of ∼20% and recurring foodborne outbreaks, listeriosis remains a significant public health concern (2, 5–7).

Disseminated infections are of particular concern in pregnant women, as Listeria monocytogenes can spread to the placenta, fetus, and/or neonate. Approximately 14% of clinically recognized cases occur during pregnancy (8). Infection may lead to pregnancy loss, preterm birth, stillbirth, and life-threatening neonatal infections (9); however, the mechanisms by which L. monocytogenes reaches and breaches the placenta are only just beginning to be understood using animal models (10). We previously established the pregnant guinea pig model of listeriosis, which mimics human disease (11). After intravenous inoculation, the maternal spleen and liver are colonized rapidly, whereas the placenta greatly resists L. monocytogenes infection and is delayed in colonization (12, 13). It is possible that the placenta can be infected only after robust dissemination of the bacteria throughout maternal organs. Alternatively, or additionally, it is possible that pregnancy-associated cases of L. monocytogenes infection represent infections caused by bacterial strains that are more virulent generally or more specifically adapted for placental colonization.

L. monocytogenes typically has a saprophytic lifestyle and is commonly found in soil, vegetation, and animal feces. Furthermore, it is highly resistant to common antibacterial precautions taken in food preparation, e.g., cold temperatures, desiccation, and high salt. These factors combine to make L. monocytogenes a common food pathogen, but the infectious dose is high, and so most cases of listeriosis are isolated, sporadic events (8). Indeed, the average adult ingests ∼10^5^ CFU four times a year, but only a small number of predisposed individuals contract listeriosis (14). Occasionally, major outbreaks occur in widely distributed foods, leading to larger numbers of infections (5, 6). It remains an open question whether these outbreak strains are more virulent than sporadic or lab reference strains.

Increasingly, we are learning about how outbreak and hypervirulent pathogenic strains arise and diverge from reference lab strains through the burgeoning field of microbial population biology. Several studies have analyzed pathogenic strains to understand their evolution and population structure (15–19), and some have assayed the virulence of representative clonal clusters relative to that of historical reference strains (20). While these studies identify molecular differences between strains that can account for their origin and altered virulence, actually assaying their virulence *in vivo* is challenging due to the large number of laboratory animals required. This is especially true when considering the testing of clinical isolates, with strains numbering in the scores or hundreds. However, the use of DNA barcodes (signature tags [STs]) can allow for multiplexed analysis of several strains within a single animal. Such studies allow researchers to understand how virulence has evolved in clinical isolates over time while comparing them to lab reference strains.

Here we characterized the virulence of 77 L. monocytogenes strains: 72 from pregnancy-associated listeriosis cases and 5 from nonpregnant immunocompromised patients. Of the 72 pregnancy-associated strains, 68 were sporadic isolates and 4 were associated with foodborne outbreaks. We set out to identify strains with increased and decreased systemic virulence compared to that of lab reference strains, using a barcode-based competition assay in pregnant- and nonpregnant-animal models. We also assayed for trends in virulence, comparing bacterial burdens across organs to determine which maternal organs were most likely to be infected in concert with the placenta.

## RESULTS

### Clinical isolates and *in vivo* screening method.

**FIG 1:**
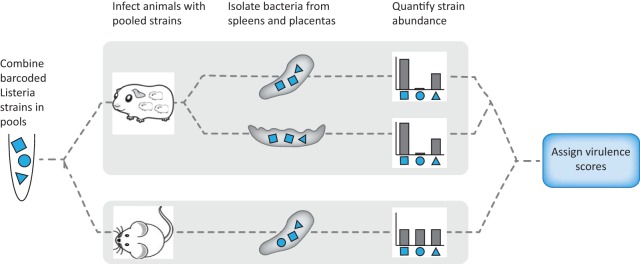
Experimental design. Signature-tagged L. monocytogenes strains were pooled and injected i.v. into pregnant guinea pigs or nonpregnant mice. Each pool contained 11 barcoded strains: 9 clinical and 2 laboratory reference strains (10403S) in the clinical strain pools and 11 laboratory reference strains in the strain 10403S pool. For each organ set (guinea pig spleen, guinea pig placenta, mouse spleen), virulence scores were assigned to each strain on the basis of the average relative abundance in the infected organs in comparison to that of the laboratory reference strains.

Our laboratory reference strain 10403S (21) is a streptomycin-resistant derivative of L. monocytogenes strain 10403, which was originally isolated from a human skin lesion in 1968 (22). 10403S is one of the most widely used strains for experimental investigation and has been passaged for decades under laboratory conditions (23). We sought to use a DNA strain barcoding and pooling assay scheme (Fig. 1) to determine how dozens of recent clinical isolates that had not been previously cultivated in the laboratory differ in virulence from 10403S.

**FIG 2:**
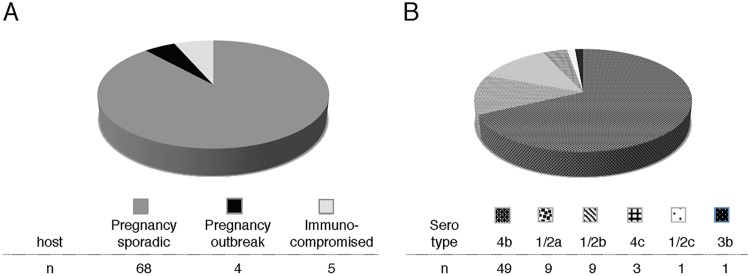
Clinical isolates. (A) Pregnancy-associated L. monocytogenes strains (*n* = 72) from 25 U.S. states were collected by the CDC between 2000 and 2010, and 5 strains were isolated from immunocompromised patients at MSKCC (*n* = 5; immunocompromised). Most of the pregnancy-associated strains were associated with sporadic cases of listeriosis and were isolated from placental tissue (*n* = 68; pregnancy, sporadic). Four strains were associated with listeriosis outbreaks in the United States (*n* = 4; pregnancy, outbreak). These 4 strains were isolated from placenta (*n* = 2), maternal blood (*n* = 1), and neonatal blood (*n* = 1). (B) Serotype distribution of pregnancy-associated strains.

We compiled 77 clinical isolates of L. monocytogenes: 72 strains from pregnancy-associated cases of listeriosis collected by the CDC over a 10-year period (2001 to 2011) in 24 U.S. states and 5 strains from the blood of immunocompromised nonpregnant patients undergoing cancer therapy at Memorial Sloan Kettering Cancer Center (MSKCC) in New York, NY (Fig. 2A; also see Table S1 in the supplemental material). Almost all strains were from sporadic cases of listeriosis. Four strains were from three different outbreaks of listeriosis associated with the following contaminated food sources: (i) Mexican-style cheese in 2005 (a placental isolate, serotype 4b) (24), (ii) turkey deli meat in 2006 (placental and neonatal blood isolates from an unrelated mother and neonate, serotype 4b) (25), and (iii) hog head cheese in 2011 (a maternal blood isolate, serotype 1/2a) (7). Only the strains from the CDC were serotyped. Among these, serotype 4b was the most common, followed by serotypes 1/2a and 1/2b, consistent with previous reports (5, 6) (Fig. 2B).

We compared the virulence of each clinical strain to that of 10403S in two animal models: (i) nonpregnant mice, the standard model for the pathogenesis of systemic listeriosis, and (ii) pregnant guinea pigs, an excellent small-animal model for pregnancy-associated listeriosis (11). In order to minimize the number of animals required for virulence screening, we incorporated a different, previously characterized DNA barcode into the chromosome of each clinical isolate (26). Clinical strains were assigned to pools *a priori*; the pools were balanced such that they included one of each signature tag from the set used, and each pool included one commonly tagged and one differentially tagged 10403S strain. Subsequently, each animal was inoculated with pools of differentially tagged bacteria. We used a total of 10 pools, each containing 11 strains marked by unique barcodes. The control pool contained 11 10403S strains, while each of the remaining nine pools consisted of 9 clinical strains and 2 10403S strains (pools A to I).

### Profiling systemic virulence in mice and guinea pigs.

**FIG 3:**
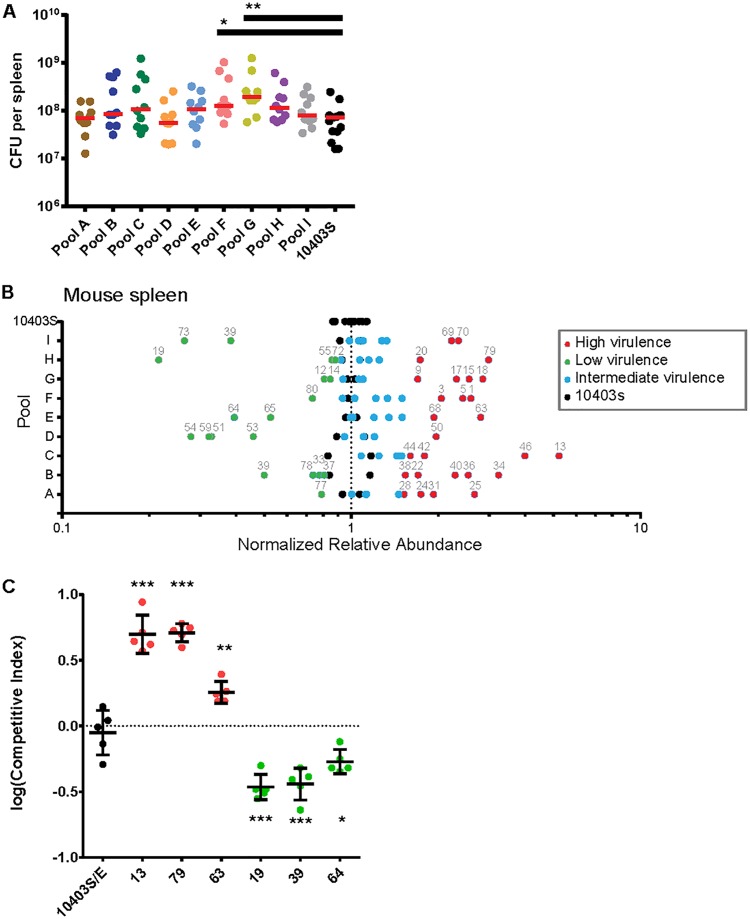
Virulence screen of clinical L. monocytogenes isolates in murine spleen. CD1 mice (nonpregnant) were infected i.v. with bacterial pools containing differentially tagged L. monocytogenes strains at equal ratios (total of 10 pools). Pools A to I contained 9 clinical strains and 2 10403S strains per pool; the 10403S pool contained 11 laboratory reference strains. Statistically significant differences in splenic bacterial burden from those in the control group were determined using one-way ANOVA with Dunnett's multiple comparisons posttest. ***, *P* < 0.0001; **, *P* < 0.01; *, *P* < 0.05. (A) Bacterial burden in murine spleen at 48 hpi with 2 × 10^5^ CFU per pool. For pools A to I there were 10 mice per pool; the 10403S pool contained 15 mice. Each circle represents the bacterial burden in one spleen, and each pool is represented by a different color. Red lines represent medians. (B) The average relative abundance of each strain in mouse spleen was quantified by qPCR. To accurately compare values across pools, the average relative abundance for each isolate was then normalized to the average for the reference strain in each pool. Significance Z-scores were calculated for the deviation from the range expected on the basis of the results for the 10403S pool (black circles). Blue circles indicate isolates with virulence similar to that of 10403S (intermediate virulence). Red and green circles indicate isolates with significantly higher and lower virulences, respectively. (C) CD1 mice were infected with one erythromycin-resistant 10403S strain and one erythromycin-susceptible untagged clinical isolate at a 1:1 ratio. The clinical isolates were chosen on the basis of their virulence scores in panel B: 3 hypervirulent (red circles) and 3 hypovirulent (green circles) strains. Competitive indices (isolate/10403S) were calculated for bacteria recovered from the spleen at 48 hpi. The control group was infected with two 10403S strains that differed in their susceptibility to erythromycin (10403S/E; black circles). Each group contained 5 mice from 2 separate experiments.

Mice were infected intravenously (i.v.) with a total of 2 × 10^5^ CFU/animal (10 animals/pool). The median bacterial burden in the control spleens at 48 h postinoculation (hpi) was 7.2 × 10^7^ CFU (Fig. 3A). The median number of CFU in the spleens of mice inoculated with pools containing clinical strains ranged from 5.6 × 10^7^ CFU (pool D) to 1.9 × 10^8^ CFU (pool G) and did not differ significantly from the median for the control pool except in two instances: the median bacterial burdens for pools F and G were 1.8- and 2.6-fold higher, respectively, than the median bacterial burden for the control pool.

Using quantitative PCR (qPCR) with primers specific for each DNA barcode, we determined the average relative abundance (RA) of each clinical strain in comparison to that of 10403S among the bacteria recovered from each spleen (Fig. 3B). We observed a range of virulence phenotypes both within and across the individually analyzed pools. We found that 27 strains were significantly more virulent (Z-score > 2.0; red points in Fig. 3B) and 18 strains were significantly less virulent (Z-score < −2.0; green points in Fig. 3B) than 10403S. Strains with significantly different virulences were present in all pools. Most pools contained one or more high- and low-virulence strains; only one pool did not contain a low-virulence strain (pool C). Importantly, four sporadic clinical strains (strains 2, 16, 21, and 39; Table S1) that were present in two different pools showed similar virulences in their two pools, suggesting that the combination of strains within each pool did not significantly influence the virulence score of individual strains.

We validated our approach by direct competition of select clinical isolates with 10403S in nonpregnant mice (27). We chose six clinical strains with virulence scores that were either significantly higher or significantly lower than the virulence score of 10403S in the pooled assay. Mice were inoculated i.v. with one clinical isolate in combination with 10403S, and their spleens were assayed for bacteria at 48 hpi. The strains differed in their susceptibility to erythromycin and were injected at a ratio of 1:1 and a total number of CFU of 2 × 10^5^/mouse. Consistent with the results of our screen, the two hypervirulent strains, strains 13 and 79, were ∼5-fold more virulent than 10430S, and strain 63 was 2-fold more virulent (Fig. 3C). In contrast, the hypovirulent strains 19, 39, and 64 were 2- to 3-fold less virulent than 10403S. These results recapitulated the virulence phenotypes identified in the screen.

**FIG 4:**
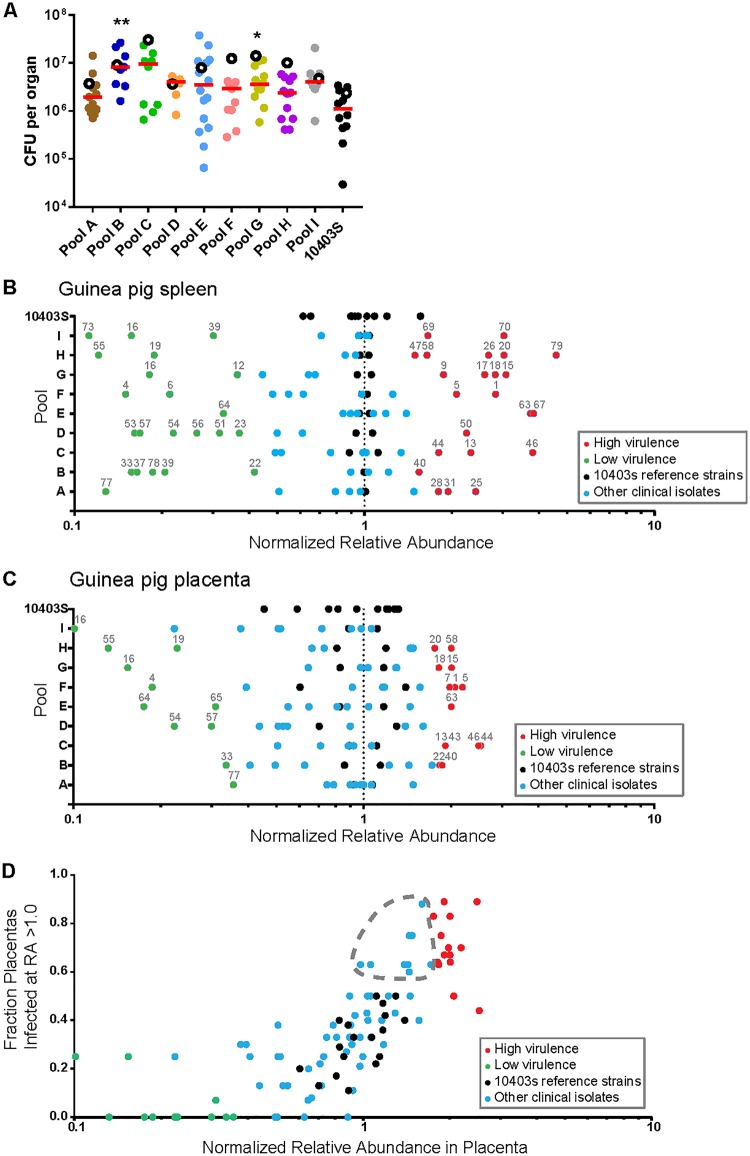
Virulence screen of clinical L. monocytogenes isolates in pregnant guinea pigs (spleen and placenta). Pregnant Hartley guinea pigs were infected i.v. with pools containing differentially tagged L. monocytogenes strains (Fig. 3). Statistically significant differences in the bacterial burden in the spleen and placenta from those in the control group were determined using one-way ANOVA with Dunnett's multiple comparisons posttest. **, *P* < 0.01; *, *P* < 0.05. (A) Bacterial burden in guinea pig spleen and placenta at 24 hpi with 10^8^ CFU per pool. The total number of guinea pigs was 27 with a total of 107 placentas. The number of placentas in each pool was as follows: pool A, 12; pool B, 8; pool C, 9; pool D, 8; pool E, 15; pool F, 10; pool G, 14; pool H, 12; pool I, 8; strain 10403S pool, 11. Each filled circle represents the bacterial burden in one placenta, and each pool is represented by a different color. Red lines represent the median number of placental CFU. Empty circles represent the median bacterial burden in spleens from each pool. (B) The average relative abundance of each strain in guinea pig spleen was quantified by qPCR, and significance Z-scores were calculated. Black dots indicate 10403S strains. Blue circles indicate isolates with virulence similar to that of 10403S (intermediate virulence). Red and green circles indicate isolates with significantly higher and lower virulences, respectively. (C) Average relative abundance of each strain in guinea pig placenta quantified and calculated as described above. (D) Correlation of the relative abundance of each strain in the placenta with the fraction of placentas that it infected at a relative abundance higher than that of its inoculant (RA > 1.0). The gray dashed outline encircles isolates that were not identified to be highly virulent by relative abundance alone but for which infected fractions comparable to those for high-virulence isolates. The color coding corresponds to that in panel C.

Next, we infected pregnant Hartley guinea pigs i.v. with 1 × 10^8^ CFU of the same pools that we used in the mouse screen and determined the bacterial burden at 24 hpi. We chose a time point earlier than the one used in the mouse screen to avoid the potentially confounding effect of bacterial trafficking between the placenta and spleen at later time points (12). Twenty-four pregnant guinea pigs were inoculated with clinical pools (2 to 5 animals/pool); 3 animals were inoculated with the control pool. The median bacterial burden in the spleens of the control pool was 2.4 × 10^6^ CFU, and the bacterial burden ranged from 3.6 × 10^6^ CFU (pool D) to 3.1 × 10^7^ CFU (pool C) in the spleens of animals inoculated with pools containing clinical isolates, indicating higher overall burdens (Fig. 4A). We determined the average relative abundance of each strain in the guinea pig spleen normalized to that of 10403S, as described above. We identified 22 hypervirulent and 20 hypovirulent strains (Fig. 4B).

In both animal models, high- and low-virulence strains were distributed stochastically across the pools, which we expected with randomized pool assignments. In the guinea pig spleen, the relative abundance of 10403S in the control pool exhibited a range wider than that in the mouse (compare Fig. 3B to 4B). However, the virulence scores of the clinical isolates were similar between mouse and guinea pig spleens. The scores were concordant for 70% (54/77) of the strains, and among the strains for which the scores were discordant, all but 1 were either hyper- or hypovirulent in one animal model and intermediately virulent in the other animal model (Table S2). Only one strain (strain 22, an outbreak strain) was hypervirulent in the murine spleen and hypovirulent in the guinea pig spleen.

### Virulence screen in the guinea pig placenta.

We evaluated the relative virulence of the clinical isolates in the placentas (*n* = 107) of the inoculated guinea pigs (8 to 15 placentas/pool). The median bacterial burden in the control group was 8.2 × 10^5^ CFU per placenta (Fig. 4A). The median for the clinical isolate pools ranged from 1.7 × 10^6^ CFU per placenta (pool A) to 8.4 × 10^6^ CFU per placenta (pool C). The range of the number of CFU across all placentas spanned 3 logs (3 × 10^4^ to 3.8 × 10^7^ CFU), which is typical for placental infection and likely due to the stringent bottleneck in placental colonization (12). Consistent with a tight bottleneck, we found the bacterial founding population in the placenta to be significantly smaller than that in the spleen. We calculated a median founding population of 1.1 × 10^5^ CFU in spleens and 278 CFU in placentas (Fig. S1).

Next, we determined the relative abundance of clinical isolates in the guinea pig placenta in comparison to that of 10403S. We identified 14 clinical strains with high virulence and 10 clinical strains with low virulence in the placenta (Fig. 4C). As in the spleen, high- and low-virulence strains were distributed stochastically across the pools. Virulence was also assayed by comparing the fraction of placentas where a strain had a high relative abundance (RA > 1) compared to its relative abundance in guinea pig placentas. We reasoned that hypervirulent strains would be able to infect more placentas as well as have a greater abundance within placentas. In general, the fraction of infected placentas did correlate strongly with the average relative abundance across placentas (Fig. 4D). However, this analysis also revealed nine strains with a fraction of infected placentas equivalent to or higher than that of several strains deemed more virulent by the relative abundance parameter described above.

Comparison of the virulence scores in the placentas and/or spleens of both rodents showed a striking degree of overlap among the three data sets. Only two strains showed a placenta-specific virulence phenotype (strains 7 and 43). These were hypervirulent in the placentas (by Z-score and fraction of infected placentas) and intermediately virulent in the spleens of guinea pigs and mice. The five strains that were isolated from immunocompromised, nonpregnant adults all had intermediate virulence scores in the placentas and various virulence scores in the spleens of both animal models (Table S1). The four outbreak strains demonstrated variable virulence scores across all organs; only one of the outbreak strains scored hypervirulent in all organs. However, due to the small number of these strains, it is not possible to draw any further conclusions.

## DISCUSSION

Here we report the *in vivo* virulence phenotypes for 77 clinical strains of L. monocytogenes: 72 from pregnancy-associated listeriosis cases and 5 from nonpregnant immunocompromised patients. Of the 72 pregnancy-associated strains, 68 were sporadic isolates and 4 were associated with foodborne outbreaks. Using a novel DNA barcode approach with qPCR, we identified isolates with either a significantly higher or a significantly lower virulence than the standard laboratory reference strain 10403S in systemic listeriosis as well as placental infection. However, no strain showed more than a 5-fold difference in virulence from that of 10403S. By using signature-tagged (barcoded) strains and qPCR, we found the 77 strains to be an even mix of hypervirulent, hypovirulent, and intermediately virulent strains. Both outbreak and sporadic clinical isolates were compared, but neither group was associated with any virulence phenotype.

Our isolates included four strains collected during recent outbreaks of foodborne listeriosis in the United States (7, 24, 25). In contrast to the bloodstream isolates from septicemic patients, these isolates were each associated with otherwise healthy pregnancies. We observed that one of these strains was highly virulent in all three assays, while the remaining three showed varied but overall moderate virulence patterns (strains 13, 21, 22, and 23; see Table S1 in the supplemental material). It is tempting to assume that outbreaks are due to increases in virulence. However, in addition to bacterial virulence, independent factors, such as the ingested dose, maternal genetics, and overall maternal health, may dramatically influence the outcome of exposure to L. monocytogenes. Evaluating the effect of any of these factors would require additional studies, potentially including prospective studies, to fully characterize the maternal status correlated with placental infection and pregnancy outcomes.

Population biology studies of pathogens have focused primarily on how virulence evolved, outbreaks arose, and antibiotic resistance spread (15–18, 28). Fewer studies have sought to compare the *in vivo* virulence of clinical strains over a period of time. In part, this is due to the high cost of animal research and the need for several animals per strain. In order to circumvent this, we developed a DNA barcode system. Previous uses of signature-tagged strains of L. monocytogenes have involved understanding bottlenecks in disseminations and alanine suppression screening to investigate virulence factors (13, 26). Here, it allowed for the simultaneous use of clinical strains in order to reduce the number of animals required to assess virulence. It has been shown previously that the insertion of signature tags via the pPL2 integration vector does not influence bacterial growth (26). Consistent with this, we did not observe any significant differences in the abundance of barcoded 10403S strains in the control pools. This technique could be even more valuable in larger, more expensive animal models, such as nonhuman primates. Additionally, the ability to test resistance to food processing techniques could be streamlined by using signature-tagged libraries of clinical strains.

We observed a larger variation in the distribution of strain abundances in the guinea pig placenta than in either of the spleen data sets. This is consistent with the previously reported bottleneck for placental infection (12, 13); therefore, we determined the founding population in the guinea pig placenta. We calculated that approximately 1/360,000 bacteria from the inoculum will infect the placenta. Many of the hypervirulent strains both had a higher abundance in the placenta and infected a greater fraction of placentas. Therefore, in assessing virulence for organs in which an infection bottleneck exists, the burden according to the number of CFU alone is an incomplete measure, and the fraction of organs infected should also be evaluated.

Clinical strains had similar virulences between the spleens and placentas. L. monocytogenes strains have been analyzed by multilocus strain typing and organized into clonal clusters (18). The most prevalent clonal clusters in bacteremia were also present in placental and neuroinvasive strains. This suggests that successful placental colonization requires a robust systemic infection. It does not mean, however, that L. monocytogenes has not evolved specialized determinants to infect the placenta. Guinea pig models have identified genes required for successful colonization of the placenta compared to the liver (29), and outbreak strains of some pathogens have been traced to novel virulence factors gained through recombination or horizontal gene transfer (30). A notable example is an enterohemorrhagic Escherichia coli O157:H7 strain that gained Shiga toxin genes via horizontal gene transfer (31). Further, Streptococcus species have novel virulence factors associated with accessory regions, that is, genes not found in the core genome (32). However, L. monocytogenes has been reported to have a highly conserved and syntenic genome (33). Out of the large number of clonal clusters from a French Listeria monocytogenes reference library, only clonal cluster 4 (CC4) strains have so far demonstrated an increase in neuronal and placental infection without an increase in splenic or hepatic infection, likely due to a novel carbon metabolism operon (20). Within our set of U.S. isolates, we observed only one instance of decreased splenic virulence and increased placental virulence. Interestingly, this strain, LS22, was isolated from neonatal blood during a deli meat outbreak (25). However, another isolate recovered from the same outbreak but isolated from a placenta (strain LS23) did not show this phenotype. Both strains were serotype 4b, which is more commonly associated with clinical cases (34).

Our lack of strains with increased placental virulence compared to virulence for maternal organs may be because our sample size of clinical isolates was ∼1/100 of that initially used by Maury et al. (20). Both studies assayed similar numbers of strains for virulence in animal models, but Maury et al. chose their strains as representatives of the starting population's clonal clusters. The tight linkage between maternal and placental virulence and the fact that human placental infection provides no epidemic selective advantage suggest that placenta-specific strains are likely rare.

Our survey of virulence in both sporadic and outbreak strains from pregnancy-associated listeriosis cases shows that U.S. L. monocytogenes isolates are evenly spread around the long-used laboratory strain 10403S, with some being more virulent and some being less virulent in animal models. This validates the use of that laboratory strain in pathogenesis studies. Further, the lack of a clear difference between outbreak and sporadic strains suggests that listerial epidemiology is not a function of pathogen virulence but is a function of other factors, likely related to individual behaviors/health and food production practices. Finally, we found a tight coupling between the maternal bacterial burden and placental infection, suggesting that a primary driver of placental susceptibility is the degree of maternal infection. The DNA barcode approach is a powerful and cost-efficient way to assess the performance of large numbers of diverse clones in animal models.

## MATERIALS AND METHODS

### Bacterial strains and culture conditions.

The laboratory reference strains were the 10403S (erythromycin-susceptible) (21), DP-L3903 (erythromycin-resistant) (27), and signature-tagged 10403S (26) strains. All L. monocytogenes clinical strains used in this study are listed in Table S1 in the supplemental material. Seventy-two clinical isolates of L. monocytogenes from pregnancy-associated listeriosis cases that occurred over 10 years (2000 to 2010) in 25 states in the United States were obtained from the Centers for Disease Control and Prevention (CDC; Atlanta, GA). Of the 72 strains, 68 (94%) were isolates from sporadic cases and 4 (6%) were from outbreaks. Five strains isolated from the blood of immunocompromised patients at Memorial Sloan-Kettering Cancer Center were a generous gift from Michael Glickman. The study was approved by the Institutional Review Board at the University of California, San Francisco, where all experiments were performed (CHR no. 11-05530). Bacteria were grown in brain heart infusion (BHI; Bacto; BD) media at 37°C. When necessary, the media were supplemented with the following antibiotics, all of which were purchased from Sigma: chloramphenicol (7.5 μg/ml), nalidixic acid (25 μg/ml), streptomycin (200 μg/ml), or erythromycin (2 μg/ml).

### Signature tag (DNA barcode) integration into clinical strains.

Unique 40-bp signature tags (STs) were inserted into the L. monocytogenes strain genomes by site-specific integration from the pPL2 vector as previously described (26). Briefly, pPL2 contains the PSA phage integrase and attachment site. This allows for stable, single-copy integration in the tRNA^Arg^ gene. The tagged clinical strains generated in this study used tags 116, 119, 191, 205, 210, 219, 231, 234, 242, 288, and 296. Integrations were confirmed by selection for chloramphenicol resistance and PCR as previously described (35). It has been shown previously that insertion of signature tags does not influence bacterial growth (26).

### Animal infections.

This study was carried out in strict accordance with the recommendations in the *Guide for the Care and Use of Laboratory Animals* of the National Research Council (36). All protocols were reviewed and approved by the Animal Care and Use Committee at the University of California, San Francisco (IACUC number AN079731-03A). Individual strains were grown in BHI at 37°C overnight. On the day of infection, 11 differentially tagged strains were combined at equal ratios to generate 10 input pools. Nine input pools (clinical pools) contained 9 clinical isolates and 2 10403S strains; one input pool (the control pool) contained 11 differentially tagged 10403S strains. Six- to 8-week-old nonpregnant female CD1 mice (Charles River Laboratories) were inoculated i.v. with a total of 2 × 10^5^ CFU pooled bacteria per animal. Pregnant Hartley guinea pigs (Elm Hill Labs, MA) were inoculated i.v. on gestational day 35 with a total of 1 × 10^8^ CFU pooled bacteria per animal. For the mouse experiments, each clinical pool was injected into five mice on two separate days for a total of 10 mice per pool; the control pool was injected into 15 mice on three separate days. Murine spleens were removed at 48 hpi. For the guinea pig experiments, each pool was injected into 2 to 5 pregnant guinea pigs, depending on the number of fetuses per dam. The total number of guinea pigs injected with clinical pools was 24 with a total of 96 placentas. The control pool was injected into 3 guinea pigs with a total of 11 placentas. Guinea pig spleens and placentas were removed at 24 hpi. Organs were homogenized in 0.2% Igepal (Sigma) with a tissue grinder. Aliquots from each output pool were plated on BHI agar plates containing 25 μg/ml nalidixic acid. The numbers of CFU per organ were enumerated, and at least 10^4^ colonies from each output pool were scraped off the plates and resuspended in phosphate-buffered saline. Aliquots of these suspensions were stored at −20°C. Input pools were prepared in the same fashion.

### qPCR.

Genomic DNA was extracted from input and output pools using a Gram-positive bacterial DNA purification kit (Epicentre), substituting mutanolysin (5 U/μl; Sigma) for lysozyme. Relative quantification by qPCR for each signature tag was achieved with previously published primer sets: signature tag-specific forward primers and the common pPL2-395R reverse primer (26). In addition, one primer set (primers LIM2 and LIMRE) was directed against *iap*, a gene used as an internal reference (37). All qPCRs were performed in a Roche LightCycler 480 qPCR machine. Each 20-μl reaction mixture contained 10 μl SsoAdvanced SYBR green universal supermix (Bio-Rad), 200 nM each primer, nuclease-free water, and template DNA. A total of 20 ng template DNA was used for experimental samples. DNA extracted from 10403S signature-tagged reference strains was used to construct qPCR standard curves for each signature tag primer set with template amounts of 100 ng, 10 ng, 1 ng, 0.1 ng, and 0.01 ng. Cycling conditions were as follows: 98°C for 2 min and 98°C for 5 s, 60°C for 20 s, and 68°C for 20 s for 40 cycles, followed by a melting curve cycle of 98°C for 15 s and 60°C for 30 s and a ramp to 98°C in intervals of 0.29°C/s. For each animal species, duplicate qPCRs for the standard curve dilutions, input and output pools, and template-free controls were run in parallel on a single 384-well plate per primer set.

The relative abundance of each signature tag in each output sample was determined in relation to that of the reference gene *iap* and the respective input pool. Quantification of cycle numbers and primer efficiencies were obtained using LightCycler software (release 1.5.0 SP3; Roche). Relative abundance (RA) values were calculated using the following equation, which accounts for different primer efficiencies (38): $\text{RA}=(({E_{\text{iap}}}^{{\text{Cq}}_{\text{iap}-\text{sample}}})/({E_{\text{ST}}}^{{\text{Cq}}_{\text{ST}-\text{sample}}}))/(({E_{\text{iap}}}^{{\text{Cq}}_{\text{iap}-\text{input}}})/({E_{\text{ST}}}^{{\text{Cq}}_{\text{ST}-\text{input}}}))$, where *E*_iap_ and *E*_ST_ are the efficiency values calculated from the standard curves for the *iap*- and ST-specific primers, respectively and *C_q_iap*-sample, *C_q_*ST-sample, *C_q_iap*-input, and *C_q_*ST-input are the quantification cycle values for the *iap* and ST samples and the *iap* and ST inputs, respectively.

### Determination of virulence.

Within each output pool, the average relative abundance was calculated for each clinical strain and divided by the average relative abundance for the two reference strains in the same output pool. This yielded an output pool-specific, normalized relative abundance for each clinical isolate. The standard deviation for the normalized abundances was calculated using the control group, which consisted of 11 differentially tagged 10403S strains. A Z-score describing the normalized relative abundance for each strain compared to that for 10403S was then calculated by subtracting the mean for the control group relative abundance and dividing by the standard deviation for the control group relative abundance. Strains that were significantly more or less abundant (*P* < 0.01) were identified according to a normal distribution of Z-scores.

### Direct competition assay.

Six- to 8-week-old female CD1 mice (Charles River Laboratories) were inoculated i.v. with 2 × 10^5^ CFU of one clinical isolate (erythromycin susceptible) and 10403S (erythromycin resistant) at a 1:1 ratio. Bacteria were recovered from the spleen at 48 hpi and enumerated, and then individual colonies were tested for differential susceptibility to erythromycin to represent the susceptibility of the clinical strain versus that of the 10403S reference strain. The control group was injected with a 1:1 ratio of two 10403S strains that differed in their susceptibility to erythromycin. Statistical significance was determined by one-way analysis of variance (ANOVA) with Dunnett's multiple comparisons posttest.

## Supplementary Material

Supplemental file 1
