# Supplementary material for: In Vivo Virulence Characterization of Pregnancy-Associated Listeria monocytogenes Infections
Source: Infect Immun. 2018 Oct 25;86(11):e00397-18. doi: 10.1128/IAI.00397-18 (PMC6204711; doi:10.1128/IAI.00397-18)
Supplement: Supplemental file 1 [file zii999092570s1.pdf]

Supplementary Information:

Table S1. Strains used in this study.

[1]Strain ID designated by outside laboratory

[1]Date received at CDC

[3]State where isolate was originally acquired

[4]As determined at CDC

[5]strain number used in this study

[6]Shading indicates significant z-scores. Red, z-score >2.0. Green, z-score <-2.0

n/a, not applicable

nd, not determined

Table S2. Comparison of splenic virulence scores between mouse and guinea pig.

Supplementary Figure S1.

Fig. S1. Quantification of the founding population in spleens and placentas of guinea pigs.

Pregnant Hartley guinea pigs (spleens n = 3; placentas n = 11) were infected i.v. with pools containing differentially-tagged in the same 10403S strain. The founding population (Nb) was calculated by the harmonic mean of the tag abundance based in the organ. A Mann-Whitney test of the the founding populations in spleens and placentas found a statistically significant difference (***P*-value = 0.0055). Filled circles represent the amount of bacteria that founded the infection of the organ in CFU/mL and red bars represent the median.

Supplemental method for estimating the founding population in each organ:

Abundance of the signature tagged bacteria was determined by using qPCR and CFU/organ data. For each signature tag in each organ, the amount of DNA in ng was calculated by a standard curve using the C_p values and known ng amounts. The frequency of abundance of signature tags was determined by dividing the calculated ng of DNA for each tag over the summed total ng for all signature tags in a given organ. The frequencies were then multiplied by the amount of CFU/organ at the time of dissection. Finally, the harmonic mean was calculated for each organ to find the effective population.

Table S1

Strain ID[1]	Date Received[2]	State[3]	Specimen Source	Serotype[4]	Signature Tag	Pool Number	Strain Source	# for this study[5]	Mouse spleen RA (wt-normalized)	GP spleen RA (wt-normalized)	GP placenta RA (wt-normalized)	Z-score mouse spleen[6]	Z-score GP spleen[6]	Z-score placenta[6]
10403S-Erm-116	n/a	n/a	n/a	1/2a	116	n/a	D. Portnoy (10403s/erm strain), this work	n/a	n/a	n/a	n/a	n/a	n/a	n/a
10403S-116	n/a	n/a	n/a	1/2a	116	1-9	Melton-Witt et al. 2012	81-89	1.044	0.993	0.905	n/a	n/a	n/a
10403S-119	n/a	n/a	n/a	1/2a	119	2	Melton-Witt et al. 2012	94	0.839	1.005	1.142	n/a	n/a	n/a
10403S-191	n/a	n/a	n/a	1/2a	191	5	Melton-Witt et al. 2012	90	1.043	0.961	1.172	n/a	n/a	n/a
10403S-205	n/a	n/a	n/a	1/2a	205	8	Melton-Witt et al. 2012	96	0.929	1.035	1.193	n/a	n/a	n/a
10403S-210	n/a	n/a	n/a	1/2a	210	7	Melton-Witt et al. 2012	97	1.031	1.056	1.171	n/a	n/a	n/a
10403S-219	n/a	n/a	n/a	1/2a	219	1	Melton-Witt et al. 2012	91	0.932	0.991	1.072	n/a	n/a	n/a
10403S-234	n/a	n/a	n/a	1/2a	234	6	Melton-Witt et al. 2012	95	1.025	1.022	1.395	n/a	n/a	n/a
10403S-242	n/a	n/a	n/a	1/2a	242	3	Melton-Witt et al. 2012	93	0.831	0.887	0.892	n/a	n/a	n/a
10403S-288	n/a	n/a	n/a	1/2a	288	4	Melton-Witt et al. 2012	92	0.890	1.066	0.703	n/a	n/a	n/a
10403S-296	n/a	n/a	n/a	1/2a	296	9	Melton-Witt et al. 2012	98	1.087	1.039	1.113	n/a	n/a	n/a
10403S-116	n/a	n/a	n/a	1/2a	116	10	Melton-Witt et al. 2012	81-89	1.126	0.906	0.946	n/a	n/a	n/a
10403S-119	n/a	n/a	n/a	1/2a	119	10	Melton-Witt et al. 2012	94	1.010	1.082	0.816	n/a	n/a	n/a
10403S-191	n/a	n/a	n/a	1/2a	191	10	Melton-Witt et al. 2012	90	0.872	0.897	1.318	n/a	n/a	n/a
10403S-205	n/a	n/a	n/a	1/2a	205	10	Melton-Witt et al. 2012	96	0.951	1.019	1.219	n/a	n/a	n/a
10403S-210	n/a	n/a	n/a	1/2a	210	10	Melton-Witt et al. 2012	97	1.065	1.194	1.302	n/a	n/a	n/a
10403S-219	n/a	n/a	n/a	1/2a	219	10	Melton-Witt et al. 2012	91	0.879	0.929	1.200	n/a	n/a	n/a
10403S-231	n/a	n/a	n/a	1/2a	231	10	Melton-Witt et al. 2012	99	0.984	1.196	0.590	n/a	n/a	n/a
10403S-234	n/a	n/a	n/a	1/2a	234	10	Melton-Witt et al. 2012	95	1.092	0.614	0.758	n/a	n/a	n/a
10403S-242	n/a	n/a	n/a	1/2a	242	10	Melton-Witt et al. 2012	93	0.863	1.560	1.119	n/a	n/a	n/a
10403S-288	n/a	n/a	n/a	1/2a	288	10	Melton-Witt et al. 2012	92	1.132	0.950	0.454	n/a	n/a	n/a
10403S-296	n/a	n/a	n/a	1/2a	296	10	Melton-Witt et al. 2012	98	1.026	0.652	1.276	n/a	n/a	n/a
2009L1022	1/22/09	IL	Placenta	1/2a	119	6	CDC and this work	1	2.593	2.834	2.063			2.950
2009L1038	2/4/09	GA	Placenta	4b	191	6	CDC and this work	2	1.335	0.482	0.394			-0.650
						9	CDC and this work		0.985	0.709	0.377			
2009L1063	2/12/09	NY	Placenta	4b	205	6	CDC and this work	3	2.049	0.979	1.564			1.479
2009L1088	3/11/09	GA	Placenta	4b	210	6	CDC and this work	4	0.933	0.150	0.187			-2.578
2009L1156	5/19/09	OR	Placenta	1/2b	219	6	CDC and this work	80	0.734	0.547	1.162			0.296
2009L1343	8/31/09	MA	Placenta	4b	231	6	CDC and this work	5	2.431	2.076	2.188			3.317
2009L1470	10/9/09	MA	Placenta	1/2c	242	6	CDC and this work	6	1.216	0.213	0.915			-0.434
2009L1496	10/21/09	MD	Placenta	4b	288	6	CDC and this work	7	1.500	1.251	1.979			2.702
2009L1554	11/10/09	NJ	Placenta	1/2b	296	6	CDC and this work	8	1.028	0.615	1.178			0.341
2009L1586	12/4/09	AL	Placenta	4b	119	7	CDC and this work	9	1.698	1.874	1.292			0.674
2010L-1640	1/15/10	NJ	Placenta	1/2b	191	7	CDC and this work	10	1.099	0.641	0.976			-0.257
2010L-1666	1/29/10	NY	Placenta	1/2b	205	7	CDC and this work	11	0.936	0.675	1.031			-0.096
2010L-1831	5/11/10	VA	Placenta	4b	219	7	CDC and this work	12	0.807	0.364	0.645			-1.229
2010L-1846	5/16/10	LA	Blood (maternal)	1/2a	234	3	Hog Head Cheese outbreak, MMWR 8 Apr 2011	13	5.232	2.327	1.911			2.497
2010L-1876	6/11/10	CT	Placenta	1/2b	231	7	CDC and this work	14	0.846	0.445	1.036			-0.079
2010L-1961	7/22/10	OH	Placenta	4b	234	7	CDC and this work	15	2.558	3.078	2.008			2.780
2010L-1991	8/4/10	TX	Placenta	4b	242	7	CDC and this work	16	1.062	0.181	0.154			-2.162
						9	CDC and this work		1.061	0.157	0.101			
2010L-2099	9/9/10	IL	Placenta	4b	288	7	CDC and this work	17	2.311	2.598	1.299			0.695
2010L-2141	9/27/10	TX	Placenta	4b	296	7	CDC and this work	18	2.852	2.825	1.815			2.212
2010L-2201	11/3/10	OK	Placenta	4b	119	8	CDC and this work	19	0.216	0.188	0.228			-2.459
2010L-2248	11/19/10	NY	Placenta	4b	191	8	CDC and this work	20	1.731	3.028	1.756			2.040
H15327	n/a	n/a	Peripheral blood	nd	119	9	Memorial Sloan-Kettering Cancer Center	69	2.222	1.654	0.981			-0.240
J0212	12/26/00	NC	Placenta	4b	210	1	NC cheese outbreak, MacDonald et al. 2005	21	1.129	0.888	0.788			-0.678
						9			1.326	1.029	0.508			
J1705	9/5/02	PA	Blood (neonatal)	4b	219	2	Turkey deli meat outbreak, Gottlieb et al. CID 2006 42:29	22	1.701	0.417	1.828			2.253
J1760	9/23/02	NJ	Placenta	4b	231	4	Turkey deli meat outbreak, Gottlieb et al. CID 2006 42:29	23	1.101	0.370	0.528			-1.573
J2685	2/13/04	NY	Placenta	4b	119	1	CDC and this work	77	0.789	0.128	0.356			-2.081
J3006	8/11/04	TX	Placenta	4b	191	1	CDC and this work	24	1.740	0.794	0.899			-0.481
J3041	9/8/04	CA	Placenta	1/2a	205	1	CDC and this work	25	2.668	2.420	1.480			1.230
J3112	10/5/04	NC	Placenta	4b	210	8	CDC and this work	26	1.070	2.679	1.458			1.163
J3215	12/21/04	NJ	Placenta	4b	219	9	CDC and this work	27	1.276	0.953	0.906			-0.461
J3245	1/7/05	NY	Placenta	4b	231	1	CDC and this work	28	1.523	1.799	0.999			-0.189
J3246	1/7/05	NY	Placenta	4b	234	1	CDC and this work	29	1.121	1.480	1.063			0.000
J3267	1/18/05	OH	Placenta	4b	242	1	CDC and this work	30	1.459	0.507	0.748			-0.926
J3306	2/10/05	NY	Placenta	4b	288	1	CDC and this work	31	1.925	1.943	0.977			-0.251
J3316	2/10/05	WI	Placenta	4b	296	1	CDC and this work	32	1.005	0.967	0.938			-0.366
J3552	8/5/05	NC	Placenta	4b	191	2	CDC and this work	33	0.776	0.157	0.336			-2.140
J3558	8/11/05	GA	Placenta	4b	205	2	CDC and this work	78	0.738	0.186	0.626			-1.285
J3668	9/23/05	NJ	Placenta	1/2a	210	2	CDC and this work	34	3.232	1.031	1.431			1.085
J3832	12/8/05	PA	Placenta	4b	219	8	CDC and this work	35	0.922	0.931	1.481			1.231
J3854	12/22/05	OH	Placenta	4b	231	2	CDC and this work	36	2.541	0.897	1.720			1.936
J3924	3/22/06	CT	Placenta	4b	234	2	CDC and this work	37	0.807	0.164	0.496			-1.667
J3975	5/12/06	CO	Placenta	1/2b	242	2	CDC and this work	38	1.538	1.209	1.225			0.478
J4027	7/17/06	IL	Placenta	4b	288	2	CDC and this work	39	0.500	0.205	0.406			-1.783
						9	CDC and this work		0.383	0.301	0.223			
J4040	7/25/06	MO	Placenta	4b	296	2	CDC and this work	40	2.285	1.543	1.863			2.355
J4080	8/17/06	CT	Placenta	1/2a	119	3	CDC and this work	41	1.082	0.492	0.710			-1.037
J4163	9/14/06	NH	Placenta	4c	191	3	CDC and this work	42	1.793	0.764	0.505			-1.642
J4456	4/6/07	TX	Placenta	4b	205	3	CDC and this work	43	1.247	1.338	1.913			2.505
J4461	4/12/07	GA	Placenta	4b	210	3	CDC and this work	44	1.601	1.802	2.530			4.320
J4551	6/21/07	CT	Placenta	4b	219	3	CDC and this work	45	1.467	0.516	0.923			-0.411
J4572	7/11/07	GA	Placenta	1/2a	231	3	CDC and this work	46	3.985	3.805	2.485			4.188
J4612	7/31/07	AZ	Placenta	4b	234	8	CDC and this work	47	1.150	1.493	1.446			1.127
J4700	8/15/07	NY	Placenta	1/2a	242	8	CDC and this work	79	2.986	4.582	0.663			-1.177
J4707	8/21/07	NY	Placenta	4b	288	3	CDC and this work	48	1.436	1.012	0.886			-0.519
J4708	8/21/07	NY	Placenta	4b	296	3	CDC and this work	49	1.492	0.986	1.065			0.008
J4834	11/5/07	GA	Placenta	4b	119	4	CDC and this work	50	1.968	2.249	1.391			0.966
J4894	12/6/07	TX	Placenta	4b	191	4	CDC and this work	51	0.329	0.316	0.546			-1.520
J4897	12/11/07	DE	Placenta	1/2b	205	4	CDC and this work	52	0.948	0.611	0.899			-0.482
J4903	12/13/07	MI	Placenta	4b	210	4	CDC and this work	53	0.458	0.161	0.438			-1.838
J4910	12/19/07	NC	Placenta	4b	219	4	CDC and this work	54	0.279	0.219	0.223			-2.471
J4969	1/31/08	TX	Placenta	4c	231	8	CDC and this work	55	0.857	0.121	0.132			-2.740
J4970	1/31/08	TX	Placenta	4b	234	4	CDC and this work	56	1.106	0.264	0.508			-1.632
J4979	2/13/08	TX	Placenta	4b	242	4	CDC and this work	57	1.202	0.168	0.299			-2.250
J5010	3/17/08	GA	Placenta	3b	288	8	CDC and this work	58	1.250	1.644	2.003			2.765
J5012	3/19/08	IL	Placenta	4c	296	4	CDC and this work	59	0.322	0.500	1.602			1.588
J5042	5/6/08	MA	Placenta	4b	119	5	CDC and this work	60	1.499	1.185	0.648			-1.218
J5150	8/7/08	VA	Placenta	4b	205	5	CDC and this work	61	0.993	1.393	0.549			-1.510
J5151	8/7/08	VA	Placenta	4b	210	5	CDC and this work	62	1.199	0.843	0.810			-0.743
J5157	8/13/08	CA	Placenta	1/2a	219	5	CDC and this work	63	2.803	3.739	2.002			2.767
J5188	8/28/08	OR	Placenta	1/2b	231	5	CDC and this work	64	0.394	0.326	0.175			-2.613
J5363	10/16/08	NY	Placenta	4b	234	5	CDC and this work	65	0.526	0.895	0.309			-2.217
J5367	10/22/08	GA	Placenta	4b	242	5	CDC and this work	66	1.014	0.944	1.040			-0.066
J5479	12/17/08	PA	Placenta	1/2b	288	5	CDC and this work	67	1.350	3.835	1.445			1.128
J5482	12/17/08	PA	Placenta	4b	296	5	CDC and this work	68	1.932	1.073	0.877			-0.546
M61909	n/a	n/a	Peripheral blood	nd	231	9	Memorial Sloan-Kettering Cancer Center	70	2.348	3.027	1.063			0.000
T21688	n/a	n/a	Peripheral blood	nd	234	9	Memorial Sloan-Kettering Cancer Center	71	1.098	1.014	0.517			-1.606
W61604	n/a	n/a	Mediport blood	nd	296	8	Memorial Sloan-Kettering Cancer Center	72	0.881	0.857	0.731			-0.978
X22426	n/a	n/a	Peripheral blood	nd	205	9	Memorial Sloan-Kettering Cancer Center	73	0.265	0.112	0.718			-1.016

Table S2

Mouse \ Guinea Pig	hypervirulent	hypovirulent	neutral
**hypervirulent**	19	1	7
**hypovirulent**	0	13	5
**neutral**	4	6	22

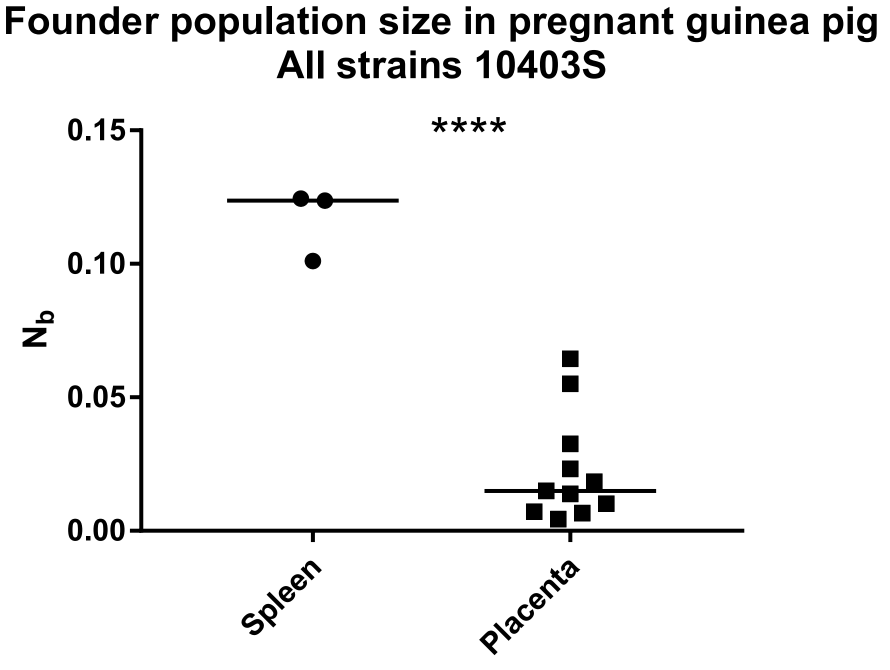

Founder population size in pregnant guinea pig
All strains 10403S
****
0.15
0.10
0.05
0.00
N$_b$
Spleen
Placenta
